# Role of RAGE and Its Ligands on Inflammatory Responses to Brain Tumors

**DOI:** 10.3389/fncel.2021.770472

**Published:** 2021-12-16

**Authors:** Griffith Kyle Otazu, Mojtaba Dayyani, Behnam Badie

**Affiliations:** Division of Neurosurgery, City of Hope Beckman Research Institute and Medical Center, Duarte, CA, United States

**Keywords:** cancer, glioma, inflammation, HMGB1, S100A8, S100A9, S100B

## Abstract

Gliomas, the most common form of brain cancer, can range from relatively slow-growing low-grade to highly aggressive glioblastoma that has a median overall survival of only 15 months despite multimodal standard therapy. Although immunotherapy with checkpoint inhibitors has significantly improved patient survival for some cancers, to date, these agents have not shown consistent efficacy against malignant gliomas. Therefore, there is a pressing need to better understand the impact of host inflammatory responses on the efficacy of emerging immunotherapy approaches for these resistant tumors. RAGE is a multi-ligand pattern recognition receptor that is activated in various inflammatory states such as diabetes, Alzheimer’s disease, cystic fibrosis, and cancer. Low levels of RAGE can be found under normal physiological conditions in neurons, immune cells, activated endothelial, and vascular smooth muscle cells, but it is over-expressed under chronic inflammation due to the accumulation of its ligands. RAGE binds to a range of damage-associated molecular pattern molecules (DAMPs) including AGEs, HMGB1, S100s, and DNA which mediate downstream cellular responses that promote tumor growth, angiogenesis, and invasion. Both *in vitro* and *in vivo* studies have shown that inhibition of RAGE signaling can disrupt inflammation and cancer progression and metastasis. Here, we will review our current understanding of the role of RAGE pathway on glioma progression and how it could be exploited to improve the efficacy of immunotherapy approaches.

## Introduction

Gliomas are classified as the most common type of brain cancer, accounting for about 80% of all diagnosed malignant brain tumors (Behnan et al., 2019). Based on the recent updates of cIMPACT−Utrecht committee on CNS tumor classification, gliomas are categorized into four grades (grade 1–4), among which grades 4s, and diffuse or anaplastic astrocytic gliomas with IDH-wildtype 4 are considered as “glioblastoma” (Gonzalez Castro and Wesseling, 2020; Louis et al., 2020). The overall age-adjusted incidence rates for all gliomas range from 4.67 to 5.73 per 100,000 persons, while for glioblastoma it ranges from 0.59 to 3.69 per 100,000 persons (Ostrom et al., 2014). For a low-grade glioma, the 10-year survival rate is 47% with a median survival time of 11.6 years (Xu et al., 2020) whereas patients with glioblastoma have a median overall survival of only 15 months (Stupp et al., 2005; Tamimi and Juweid, 2017).

Most patients with gliomas undergo surgical resection of the tumor, followed by radiation and chemotherapy (Davis, 2016; Banerjee et al., 2021). The goal of the surgery is to remove most of tumor without damaging the surrounding normal brain tissue. For low-grade gliomas, the extent of resection has been associated with better overall survival (Young et al., 2015; Morshed et al., 2019); but for higher-grade gliomas, survival benefit from aggressive surgery is modest (Young et al., 2015). Following surgery, patients are then treated with radiation and chemotherapy (typically temozolomide) as an adjuvant treatment (Banerjee et al., 2021; Singh et al., 2021). While advancements in these therapies have marginally extended the overall survival for patients with glioblastoma, tumor relapse is inevitable. A number of factors account for glioblastoma resistance to standard therapies. These include: tumor invasion into normal brain tissue, resistance to chemo/radiation therapy, molecular heterogeneity, and immunosuppressive stromal cells that promote evasion of host anti-tumor immune responses (Razavi et al., 2016; Medikonda et al., 2021).

Among the glioma-associated stromal cells, infiltrating microglia (MG) and macrophages (MP), (also known as tumor-associated macrophages or TAMs) are well known due to their involvement in glioma escape from anti-angiogenic agents (Piao et al., 2012; Chaudhary et al., 2021). TAMs, as a component of the innate immune system, are derived from both resident brain MG and myeloid-derived monocytes. A wide range of pattern recognition receptors that are expressed by TAMs, continuously monitor changes in tumor microenvironment. Among these, receptor for advanced glycation end-products (RAGE) is known as a membrane protein that binds glycosylated macromolecules and implicated in various human diseases (Ramasamy et al., 2009; Sorci et al., 2013; Shen et al., 2020). For instance, in conditions like diabetes, chronic inflammation or neurodegenerative disorders, RAGE expression is increased dramatically in vasculature, hematopoietic cells, and the central nervous system (CNS), while in normal physiological conditions it is expressed at high levels in the lungs, and at lower levels in different cell types including neurons, immune cells, activated endothelial and vascular smooth muscle cells (Sorci et al., 2013). Coupling of RAGE by its ligands activates several downstream signaling cascades that ultimately lead to the upregulation of cytokines, chemokines, adhesion molecules, and other molecular pathways involved in cell proliferation, differentiation, migration, survival, phagocytosis, and authophagy via the activation of NF-κB, ERK1/2, p38 and STAT3 (Sorci et al., 2013; Erusalimsky, 2021). Activation of these downstream regulatory pathways is one reason that RAGE signaling is considered to be involved in tumorigenesis. In this review, we provide an overview of the role of RAGE and its ligands on inflammatory responses to gliomas.

## Inflammation in Glioma

Glioblastoma is known to foster an inflammatory immune response that could shift the tumor microenvironment into a pro-tumorigenic milieu (Fan et al., 2020). Acute and transient inflammation may inhibit tumor growth (Fan et al., 2020) by upregulating pro-inflammatory cytokines such as TNFα, IL-1β, and IL-6 that are part of the initial inflammatory cascade and recruit other downstream targets to enhance anti-tumor responses. However, if inflammation becomes a chronic occurrence, the same inflammatory processes can exhaust the immune system’s ability to fend off glioma cells. These complex microenvironmental forces, combined with mutations in tumor oncogenes create a tumor milieu that can evade immune recognition that spreads throughout the brain parenchyma. While the presence of pro-inflammatory cytokines clearly affects the immune system in gliomas, their expression also contributes to the development of various neoplastic processes. For example, TNFα has been implicated as an inducer of angiogenesis, mainly through EGFR upregulation (Al-Kharboosh et al., 2020). Also, TNFα has been shown to facilitate glioma invasion, not by upregulating MMP2 or MMP9, but through modulation of MEK-ERK1/2 pathway (Ramaswamy et al., 2019). Similarly, increased expression of both IL-1β and IL-6 has been correlated with poorer survival rates in patients with glioblastoma (Al-Kharboosh et al., 2020), possibly through activation of STAT3 signaling and/or, NF-κB pathway. TNFα and IL-1β are known activators of NF-κB, and promote downstream inflammatory pathways by inducing NLRP3 inflammasome (Liu et al., 2017). IL-6, on the other hand, is not an activator of NF-κB, but is a cytokine released upon NF-κB activation (Mostofa et al., 2017). These complex inflammatory responses, although well-characterized in other inflammatory or pathological conditions, continue to be analyzed in glioblastoma models in order to improve the efficacy of immunotherapy approaches. The goal of this review is to examine how RAGE and its ligands alter glioma inflammatory responses and anti-tumor immune response.

## RAGE

RAGE is a multiligand receptor that is heavily involved in inflammatory responses. This receptor is classified as a transmembrane protein that belongs to the immunoglobulin (Ig) superfamily (Mulrennan et al., 2015). RAGE is also considered a pattern recognition receptor (PRR) due to its ability to recognize multiple classes of molecules (Chavakis et al., 2004). The most well-categorized ligands that interact with RAGE are AGEs, HMGB1, and members of the S100 family (Mulrennan et al., 2015), and modulate a range of immunological and inflammatory signaling pathways by activating RAGE.

### Localization

RAGE is typically expressed on membranes of immune cells such as monocytes/macrophages, neutrophils, lymphocytes, and dendritic cells (Chuah et al., 2013). Additionally, RAGE is observed on fully differentiated neurons, and prolongs their survival during embryonic development (Lee and Park, 2013). During embryogenesis, the expression of both RAGE and its ligands is the highest, but tapers off to baseline levels in mature cells. The other well-characterized instance of RAGE activation is in chronic inflammation (Schmidt et al., 2001), which underscores its role as an inflammatory modulator.

### Isoforms (Splice Variants)

RAGE receptors are composed of three subunits: an extracellular region that contains a V-type region that interacts with ligands, a transmembrane region, and a cytoplasmic region that allows signal transduction in the cell (Lee and Park, 2013). In addition to full-length RAGE, there are RAGE variants that are generated through different splicing patterns. These variants include N-truncated, dominant-negative, and soluble RAGE. The soluble RAGE variant (sRAGE) is of particular therapeutic interest. This isoform arises through either cleavage by proteases or by mRNA splicing (Chuah et al., 2013). As a result, sRAGE lacks a transmembrane domain and is capable of detaching from the cell surface but still contains its ligand-binding V-domain which allows it to potentially bind RAGE ligands and antagonize RAGE activation (Lee and Park, 2013).

### RAGE and Neuroinflammation

**FIGURE 1:**
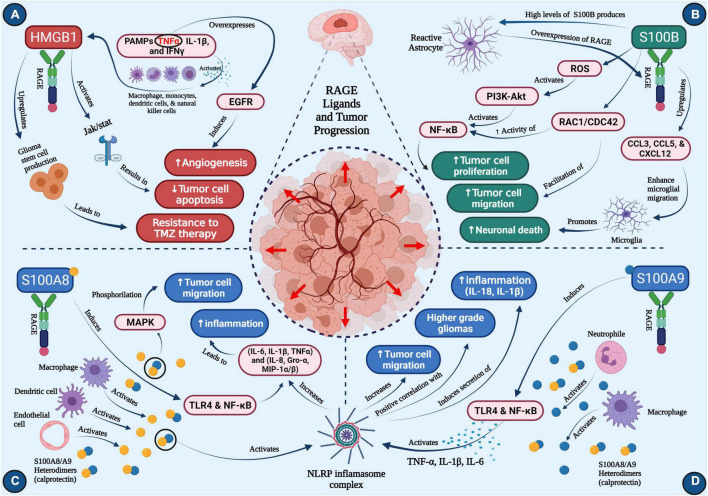
Roles of RAGE ligands in glioma progression **(A)** HMGB1, **(B)** S100B, **(C)** S100A8, **(D)** S100A9. Concept map depicts the major roles of four critical ligands of RAGE on glioma progression. Each section depicts pathways that activate downstream cascades important in neuro-inflammation, tumor cell migration, neuronal death, angiogenesis, temozolomide resistance, ultimately resulting in tumor progression (created with BioRender.com).

RAGE is well-positioned to influence both inflammatory responses (due to its expression on a wide array of immune cells) and signaling processes in the CNS as a result of its localization in neurons and their proximity to other cell types within the brain. One common characteristic of RAGE and its ligands is that they participate in a positive feedback loop where upregulation of ligands will stimulate greater expression of RAGE receptors, thereby potentiating receptor-ligand interactions (Schmidt et al., 1999). This positive feedback loop has been observed in neurons expressing RAGE, and in turn leads to the activation of ERK1/2 (Kierdorf and Fritz, 2013). Furthermore, RAGE engagement can lead to the activation of the MAPK pathway and the production of reactive oxygen species (ROS), which are known to cause cell death. In examining Jurkat E6 leukemia cells and blood-derived mononuclear cells (monocytes, macrophages, and dendritic cells), one of the earlier studies found that high levels of S100B (RAGE ligand secreted by reactive astrocytes) potentiates RAGE expression and triggers the release of pro-inflammatory cytokines like TNFα and IL-1β (Hofmann et al., 1999; Figure 1).

### RAGE as an Alarmin Detector

The most well-known ligands of RAGE are often characterized as a damage-associated molecular patterns or DAMP proteins, which alert the immune system of infection, cellular damage, or other injury (Roh and Sohn, 2018). In contrast to pathogen-associated molecular patterns (PAMPs) RAGE ligands are endogenous signals and do not stem from outside the body. As DAMPs, the role of RAGE ligands in inflammation is well-known, but in gliomas, their role continues to be elucidated. As mentioned, gliomas exploit the host inflammatory responses to promote their migration/progression, and thus, understanding the role of each RAGE ligand on tumor microenvironment will be critical in developing more effective therapies.

## RAGE Ligands

### High-Mobility Group Box 1

The high-mobility group box 1 (HMGB1) protein belongs to the HMGB superfamily and is expressed within the nucleus as a mediator of transcriptional processes. Additionally, HMGB1 is found throughout the cytoplasm where it modulates autophagy (Magna and Pisetsky, 2014). However, its interaction with RAGE becomes most pertinent upon its translocation outside the cell. Extracellular HMGB1 assumes the role of a DAMP molecule (Harris et al., 2012), and in doing so, becomes a key regulator of immune processes through upregulation of pro-inflammatory cytokines and chemokines.

HMGB1 translocation typically occurs in the context of inflammation or immunogenicity, and is linked to disease states in which these processes are common. More specifically, HMGB1 is known to be released into extracellular space upon the body’s immune response to foreign agents (Andersson and Tracey, 2011). One well-characterized route that enables this translocation is the activation of the innate immune cells, such as macrophages, monocytes, dendritic cells, and natural killer (NK) cells, by the sensing of PAMPs or pro-inflammatory cytokines like TNFα, IL-1β, and IFNγ (Figure 1A). LPS is another stimulant that is capable of inducing prolonged secretion of HMGB1 into extracellular space, following an increase in HMGB1 mRNA expression (Andersson and Tracey, 2011; Magna and Pisetsky, 2014).

The other mechanism of HMGB1 translocation into the extracellular space is through cell death (Yang et al., 2020), however, the specific mechanism of cell death is important in this process. In necrosis, HMGB1 can be passively released and displays a heightened mobility within the nucleus that, when paired with its relatively weak bonding with chromatin, enabled HMGB1 to relocate to the outside of the cell (Scaffidi et al., 2002; Xue et al., 2021). Conversely, the opposite may occur in apoptosis where HMGB1 intranuclear mobility is suppressed (Scaffidi et al., 2002). Based on these observations, it seems that the increased presence of HMGB1 through translocation may serve as a more reliable indicator of pathogenic processes since such events are usually characterized by necrosis instead of apoptosis (which is more commonly associated with physiological responses). For cells that do not express HMGB1, their death does not stimulate the production of inflammatory cytokines (Rovere-Querini et al., 2004). These observations suggest that HMGB1 plays a crucial role within immune responses due to its ability to mediate inflammation in the context of necrosis, a hallmark of glioblastoma.

Once HMGB1 has translocated to extracellular space, it exerts its tumorigenic properties. While well characterized in its ability to bind to RAGE, HMGB1 can also interact with TLR-2, TLR-4, and TLR-9 (Magna and Pisetsky, 2014; Xue et al., 2021). The RAGE-HMGB1 axis is a major aspect of immune signaling in pathogenic conditions like glioma and inflammatory diseases. However, by binding to multiple toll-like receptors, HMGB1 can mediate a greater number of pro-inflammatory pathways. For example, TLR-4 engagement activates TNF-α release from macrophages (Yang et al., 2010; Xue et al., 2021). Another HMGB1 signaling pathway is the activation of JAK/STAT (Figure 1A), which enables modulation of cell growth and differentiation, and migration of immune cells (Seidu et al., 2017). Increased expression of STAT has been known to inhibit apoptosis of tumor cells, and therefore, HMGB1 activation of STAT is considered pro-tumorigenic. Other potential downstream HMGB1 targets include MAPK, p38, and NF-κB (Xue et al., 2021). HMGB1 not only induces the release of pro-inflammatory cytokines, but synergizes with LPS by binding to RAGE (Qin et al., 2009; Rauvala and Rouhiainen, 2010).

Due to its implications in neuro-inflammation, HMGB1 has been considered as a therapeutic target in gliomas. Hong et al. (2019) examined the effects of HMGB1 on the efficacy of oncolytic herpes simplex virus (oHSV), and demonstrated that the use of HMGB1-blocking antibodies increased the survival of glioma-bearing animals. Additionally, they analyzed the activation of tumor endothelial cells by showing significant decrease in their permeability with anti-HMGB1 antibodies. Since endothelial cell activation is positively correlated with cerebral edema, they observed a decrease in cerebral edema in mice treated with both oHSV and anti-HMGB1 antibodies compared to mice treated with a control antibody.

HMGB1 has also been implicated as a key mediator of glioma resistance to TMZ (Gao et al., 2021). As previously mentioned, TMZ resistance is a common setback in treating patients with malignant gliomas and is an inherent characteristic that ultimately drives relapse of primary neoplasms. A well-characterized mechanism by which treatment resistance occurs is through the production and differentiation of glioma stem cells (GSCs). Classified as tumor-initiating cells, GSCs can not only stimulate tumor growth, but they may also contribute to treatment failure due to their resistance to both drugs and radiotherapy (Auffinger et al., 2014; Garnier et al., 2018). Further studies revealed that HMGB1 upregulates GSC production, and thereby resistance to TMZ, via activation of the Wnt/β-catenin pathway (Gao et al., 2021). This work suggests that successful targeting of HMGB1 may improve glioma treatment responses to TMZ (Figure 1A).

### S100B

The S100 protein family consists of 25 unique members, each of which contributes to a multitude of processes ranging from maintenance of calcium balance and cell growth to migration (Chen et al., 2014; Allgöwer et al., 2020). However, in the context of this review, the behavior of S100 proteins as cytokine-like agents is of particular interest, especially within RAGE signaling pathways. RAGE interactions with S100B and S100A9 are well characterized within glioma, but in addition to these two proteins, 11 other S100 members have also been demonstrated to interact with RAGE (Leclerc et al., 2009).

According to The Human Protein Atlas, the brain is one of a few organs that exhibits high localization of S100B (Donato et al., 2013). At basal physiological states, S100B, has been shown to be neuroprotective. However, increased levels of S100B have been shown to upregulate pro-inflammatory pathways that can play a role in the pathogenesis of not only gliomas but also Alzheimer’s and epilepsy (Bianchi et al., 2011). Elevated S100B concentrations are typically caused by brain injury, which leads to subsequent release of S100B into extracellular space. As a result, S100B has been proposed to act as a DAMP (Villarreal et al., 2014), much like HMGB1.

RAGE-S100B interaction can activate the PI3K-AKT signaling pathway (Allgöwer et al., 2020), thus improving survival of neuroblastoma cell (Leclerc et al., 2007). In microglia, NF-κB (an essential regulator of inflammatory responses) is also a downstream target of S100B that is mediated by RAGE (Figure 1B), suggesting its role in promoting neuro-inflammation following brain injury (Leclerc et al., 2009). Finally, S100B upregulates CCL3, CCL5, and CXCL12 chemokines in a RAGE-dependent manner (Bianchi et al., 2011; Figure 1B), which then increases microglia trafficking into the brain.

As previously mentioned, astrocytes are a primary producer of S100B, and as such, may be involved in the deregulation of S100B expression and activity in the tumor-adjacent brain or gliomas. As tumors that arise from astrocytes, most astrocytomas express high levels of S100B. Cultured astrocytes have been shown to harness a variety of molecular pathways upon exposure to S100B. Both NF-κB and AKT were activated, but so were other RAGE-dependent pathways like Rac1 and CDC42 (Villarreal et al., 2014). These two proteins were required for S100B-mediated to cause astroglial stellation. The outgrowth of astrocytic cells then enabled cell motility, as evidenced by increased invasion in a wound-healing assay.

The link between S100B and glioma is being investigated by our group. One of our first studies that analyzed S100B in relation to gliomas, found that S100B modulated STAT3 activity in microglia (Zhang et al., 2011; Wang et al., 2013). Also, S100B fostered a cellular microenvironment that upregulates the CCL2 chemokine, allowing for migration of TAMs into gliomas (Wang et al., 2013). We also confirmed that increased S100B expression led to RAGE upregulation, thus confirming the presence of a positive feedback loop between RAGE and its ligands in gliomas. Further studies by our group showed that inhibiting S100B via administration of duloxetine (an SSNRI) abrogated glioma growth and reduced TAM infiltration (Gao et al., 2018). In addition, it has been demonstrated that poor prognosis in recurrent glioma patients correlates with high S100B levels (Holla et al., 2016). Together, these findings suggest S100B to be an important modulator of glioblastoma microenvironment and potentially be used as a therapeutic target.

### S100A8/S100A9

S100A8 and S100A9 are another pair of members of the S100 calcium-binding family that, like S100B, are characterized as common RAGE ligands. These two S100 subtypes exist predominantly in a heterodimeric form (which is also known as calprotectin) upon release from neutrophils and monocytes (Wang et al., 2018). The S100A8/S100A9 heterodimer form is much more stable than S100A8 and S100A9 alone, however, it has also been observed that S100A9 homodimers are able to form in inflammatory environments and maintain their role in regulating inflammation (Riva et al., 2013; Mondet et al., 2021).

Both S100A8 and S100A9 are constitutively expressed by myeloid cell types, and may act as alarmins upon onset of pathological conditions, which include secretion by necrotic cells or activated immune cells (Crowe et al., 2019). S100A8 activation is also observed in cells involved in inflammatory responses, namely macrophages, dendritic cells, and endothelial cells (Wang et al., 2018; Figure 1C). Similarly, S100A9 is highly expressed in neutrophils and macrophages (Figure 1D). Interestingly, S100A8 appears to foster myeloid cell differentiation and inhibit telomerase activity, but S100A9 actually opposes the S100A8-induced reduction in telomerase activity, in addition to inhibiting myeloid differentiation (Donato et al., 2013; Mondet et al., 2021). This implies a potential regulatory interaction between these two proteins in pathological conditions.

One established downstream mediator of S100A8/S100A9 signaling is MAPK, which upon phosphorylation can induce tumor cell migration (Srikrishna, 2012); this is of particular interest because S100A8 is a chemotactic agent. Much like S100B, these two S100A subtypes are also integrated into the inflammatory response as DAMP molecules. The regulation of pro-inflammatory cytokine release is also well established, as S100A8 and S100A9 bind to TLR4 and activate NF-κB (Mondet et al., 2021). S100A8/S100A9 are also implicated in the activation of the NLRP3 inflammasome (Simard et al., 2013), which has been shown to play a role in glioma progression.

The S100A8/S100A9 subtypes may be promising biomarkers for glioblastoma. Not only were transcript levels of these genes upregulated in higher-grade gliomas, but overall survival rates were also lower in patients with greater expression of either form (Kan et al., 2020). Further studies have corroborated the presence of increased S100A8 and S100A9 levels in glioblastoma (Popescu et al., 2014; Arora et al., 2019). However, despite showing greater expression, serum levels of S100A9 were not a sufficient prognostic measure of glioblastoma at higher transcript levels, while only S100A8 maintained a significant correlation as a marker (Arora et al., 2019). One potential limitation that may affect the utility of S100A8 as a glioblastoma marker is its overexpression in other neurological and inflammatory diseases (Arora et al., 2019).

As alluded to previously, S100A8/S100A9 are implicated in the activation of NLRP3 inflammasome. The inflammasome unit is known to respond to various pathological conditions, such as bacterial and viral infections and DAMP-mediated inflammation (Swanson et al., 2019). Within inflammatory responses, NLRP3’s role centers around inducing the maturation and subsequent secretion of pro-inflammatory cytokines like IL-1β and IL-18 (Lamkanfi and Dixit, 2014). Analysis of tumor protein expression revealed a significantly positive correlation between NLRP3 levels and WHO glioma grade (Yin et al., 2018). Activation of NLRP3 expression by S100A8/S100A9 inhuman PBMCs increased the secretion of pro-inflammatory cytokines (IL-6, IL-1β, TNFα) and chemokines (IL-8, Gro-α, MIP-1α/β) that are prevalent in gliomas (Simard et al., 2013). Further research into the interactions between S100A8/S100A9, NLRP3, and common inflammatory cytokines may provide potential therapeutic targets for gliomas (Figures 1C,D).

## RAGE and Immunotherapy

Glioblastomas, in general, are considered to be immunologically “cold” tumors and not prone to immunotherapy. Although immunotherapies have improved the management of many solid tumors and hematologic malignancies, phase III studies of PD-1 checkpoint inhibition in both newly diagnosed and recurrent glioblastoma patients showed no efficacy (Reardon et al., 2020). Factors that have been proposed for glioblastoma resistance to checkpoint inhibitors include: high tumor heterogeneity, low mutational burden, systemic immunosuppression, and local immune dysfunction (Razavi et al., 2016; Medikonda et al., 2021). One of the hallmarks of the glioblastoma local immune dysfunction is T cell exhaustion, in which recurrent or prolonged antigen exposure impair T cell anti-tumor function (Medikonda et al., 2021). Furthermore, glioblastomas upregulate multiple immune checkpoints such as PD-1, TIM-3, LAG-3, and CTLA-4, that facilitate T cell exhaustion (Woroniecka et al., 2018). Also, recent evidence indicates a relation between RAGE ligands and T cell exhaustion. In a polytrauma rat model study investigating the association of HMGB1 levels in mediating dysregulated immune responses and its effects on the cellular levels of RAGE and toll-like receptor 4 (TLR4); it has been revealed that HMGB1 surge is responsible for the onset of T cell exhaustion and dysfunction, resulting in diminished RAGE and TLR4 surface expression and possibly preventing T cell function (Muire et al., 2021).

**FIGURE 2:**
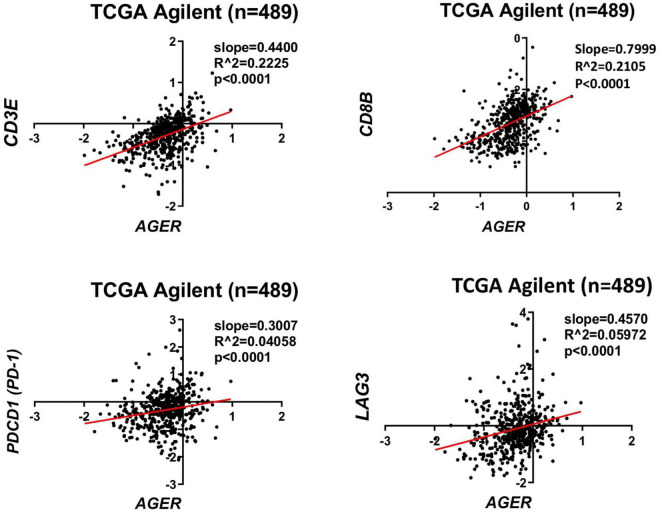
Correlation of RAGE expression to inflammatory markers in glioblastoma. TCGA analysis confirms a direct correlation between CD3 and CD8 lymphocyte markers with *AGER*. Expression of T cell exhaustion markers PD-1 and LAG3 also correlates with AGER expression.

**FIGURE 3:**
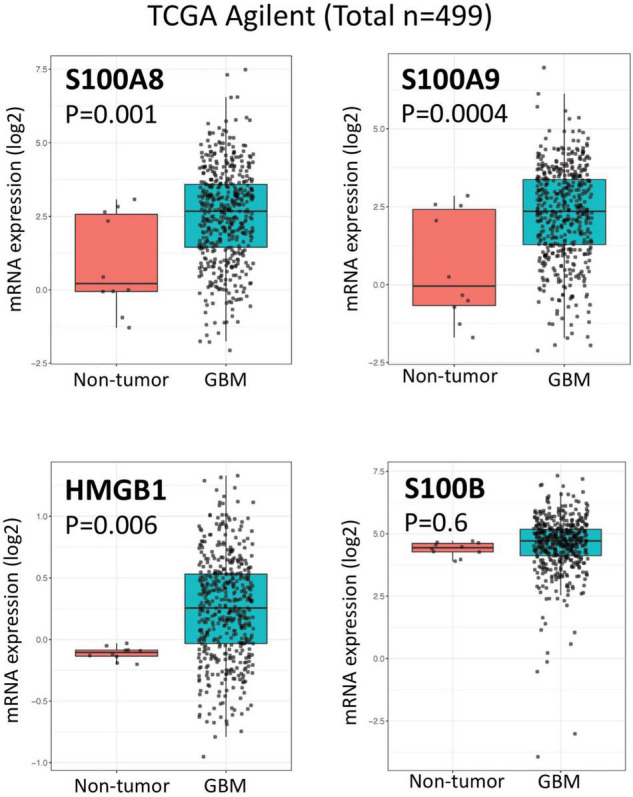
Expression of RAGE ligands in glioblastoma and non-tumor samples. Box plots of TCGA analysis demonstrate significant expression of RAGE ligands (HMGB1, S100A8, and S100A9) in glioblastoma.

Considering the role of RAGE and its ligands on promoting a pro-inflammatory tumor microenvironment, it’s likely that alterations of RAGE signaling pathway may also impact the efficacy of immunotherapies for malignant gliomas. Our analysis of the human glioma TCGA database (Squatrito, 2015–2021) suggest a direct correlation between *AGER* (RAGE gene) and a number of lymphocyte markers in glioblastoma (Figure 2). Although the expression of CD3, CD8, appear to directly correlate with AGER, the expression of T cell inhibitory genes, such as PD-1 and LAG3 were also higher in RAGE-over-expressing glioblastomas. This finding confirms that RAGE activation does indeed promote a pro-inflammatory immune environment, but at the same time, may be associated with T cell exhaustion. Also, expression of RAGE ligands in tumor and non-tumor brain tissue in TCGA database revealed substantial expression of RAGE ligands in glioblastoma (Figure 3). Whether RAGE inhibition could impact the efficacy of immunotherapies by altering T cell trafficking and/or exhaustion needs further investigation.

Although the efficacy of small molecule RAGE inhibitors on immunotherapy responses have not been studied yet, others have suggested that such an approach may be reasonable. For example, tasquinimod, a second-generation oral quinoline-3-carboxamide that has been shown to bind S100A9 in the tumor microenvironment, blocks engagement of S100A9 with RAGE, and thus, prevents activation of downstream signaling pathways that lead to production of pro-inflammatory cytokines (Kierdorf and Fritz, 2013; Chen et al., 2014; Wang et al., 2018). Although the anti-tumor effects of tasquinimod were initially documented in a wide range of preclinical tumor models (Isaacs et al., 2006; Jennbacken et al., 2012), phase II and III trials in metastatic prostate cancer, hepatocellular, ovarian, gastric, and renal cell carcinomas showed no clinical benefit as a monotherapy (Bratt et al., 2009; Pili et al., 2011; Sternberg et al., 2016; Escudier et al., 2017). Others, however, have shown tasquinimod to block the trafficking of myeloid derived suppressive cells (which inhibit the anti-tumor activity of CD8 and CD4 cells) (Raymond et al., 2014), change the polarization of tumor-associated macrophages into M1 phenotype (Olsson et al., 2015), and improve efficacy of PD-L1 blockade (Shen et al., 2015; Nakhle et al., 2016). These findings support further evaluation of RAGE and RAGE inhibitors as immune modulators for glioblastoma immunotherapy.

## Conclusion

RAGE signaling has been implicated in the pathogenesis of a variety of cancers including gliomas. Activation of RAGE by a plethora of ligands can modulate cellular properties that are involved in tumor proliferation, angiogenesis, invasion, metastasis and immune modulation. Thus, RAGE could be an attractive therapeutic target for many cancers, especially those that harness multiple oncogenic pathways for growth and invasion. Although small molecule RAGE inhibitors have been developed and tested for treatment of Alzheimer’s disease, these inhibitors have not been critically evaluated in glioblastoma models. Available data suggest that such inhibitors may have clinical utility when used alone or in combination with other glioma therapies.

## Author Contributions

GO: literature search, drafting of the manuscript and final approval of the version to be published. MD: critical revision of the content for intellectual material, designing the concept map, and final approval of the version to be published. BB: conception, critical revision of the content for intellectual material, supervision, and final approval of the version to be published. All authors contributed to the article and approved the submitted version.

## Conflict of Interest

The authors declare that the research was conducted in the absence of any commercial or financial relationships that could be construed as a potential conflict of interest.

## Publisher’s Note

All claims expressed in this article are solely those of the authors and do not necessarily represent those of their affiliated organizations, or those of the publisher, the editors and the reviewers. Any product that may be evaluated in this article, or claim that may be made by its manufacturer, is not guaranteed or endorsed by the publisher.

## Abbreviations

AGE: Advanced glycation end products
AKT: Protein kinase B
CNS: central nervous system
DAMPs: damage-associated molecular pattern molecules
ERK: Extracellular-signal-regulated kinase
HMGB1: high-mobility group box 1
JAK: Janus kinase
MG: microglia
MP: macrophages
MAPK: Mitogen-activated protein kinase
NK: natural killer
NF-kB: Nuclear factor kappa B
oHSV: oncolytic herpes simplex virus
PAMPs: pathogen-associated molecular patterns
PD-L1: Programmed death-ligand 1
PRR: pattern recognition receptor
PI3K: Phosphoinositide 3-kinases
RAGE: receptor for advanced glycation end-products
ROS: reactive oxygen species
sRAGE: soluble RAGE
SSNRI: selective serotonin and norepinephrine reuptake inhibitors
TAMs: tumor-associated macrophages
TLRs: Toll-like receptors
TMZ: temozolomide.
